# Characterization of Urban Particulate Matter by Diffusive Gradients in Thin Film Technique

**DOI:** 10.1155/2018/9698710

**Published:** 2018-02-05

**Authors:** Michaela Dufka, Bohumil Dočekal

**Affiliations:** Institute of Analytical Chemistry of the Czech Academy of Sciences, v.v.i., Veveří 97, 602 00 Brno, Czech Republic

## Abstract

A diffusive gradient in thin films (DGT) technique was employed in characterization of the particulate matter related to the urban area suffering from heavy traffic. Kinetics of mobilization metals fluxes from the metal-contaminated particulate matter was investigated. To monitor responses of the particulate matter sample, DGT probes of various thickness of diffusion layer were deployed in aqueous model suspensions of the particulate matter for different time periods. Particulate matter samples and exposed DGT resin gels were decomposed in a mixture of nitric and hydrochloric acid in a microwave pressurized PTFE-lined system. Total content of some traffic-related elements (Cd, Co, Cu, Mo, Ni, Pb, Pd, Pt, Rh, Sb, and V) was determined by inductively coupled plasma mass spectrometry. DGT measurements revealed that two metals pools associated with particles could be recognized, which can be characterized as high soluble fraction and almost insoluble fraction. DGT-measured metal fluxes from the labile pool showed significant difference in mobilization and resupply fluxes of individual selected elements, which might reflect the origin of selected metals and their speciation in particulate matter. The DGT technique can be applied as a useful tool for characterization of metals mobilization from the particulate matter.

## 1. Introduction

The particulate matter (PM) has received growing attention since many epidemiological studies demonstrated an association between exposure to particles and adverse human health effects [1, 2]. Particulate pollution encompasses emissions from both natural and man-made sources. The main source of PM in urban areas is road transport in addition to the burning of fossil fuels in power stations and factories. The particulate matter emitted from on-road motor vehicles includes complex mixtures of metals from tires, brakes, parts wear, engine emissions, and dust from road surfaces. Fine particulate material containing platinum group elements (PGE) is also introduced into the environment due to utilization of three-way catalytic converters [3].

The PM comprises a mixture of numerous components, including various organic substances, elemental carbon, and inorganic substances. The metallic fraction is associated with several adverse health effects. The content of transition metals in PM is significantly affected by vehicle volume and speed, type of engine and its operation conditions, road type, rush time periods, neighboring environment and meteorological conditions, etc. [4, 5]. As urban traffic is continuously increasing, traffic-generated atmospheric pollutant loads will impose an even greater impact on human and ecosystem health. Epidemiological studies pointed out a number of adverse health effects associated with atmospheric particles originating from transportation [6–8].

Conventional analytical metal measurements in atmospheric particles usually entail the determination of total metal concentrations. Nevertheless, it is often necessary to quantify specific metallic forms, species, since bioavailability, solubility, geochemical transport, and metal cycles largely depend on physical-chemical speciation [9]. Consequently, toxicity of transition metals associated with atmospheric PM depends on solubility [10], speciation, and kinetics of metal release from the solid phase. The use of water as a leaching agent was recommended [9] because these conditions are more similar to environmental conditions of extraction by rain water or to conditions in the lung environment, when PM is inhaled.

In recent decades, batchwise equilibrium-based single [11] or sequential extraction schemes [9] have been consolidated as analytical tools for fractionation analysis to assess the ecotoxicological significance of metal ions in solid environmental samples. However, they cannot simply provide information about the kinetics of the leaching process. Moreover, the ratio of the solid phase mass to volume of leaching agent, particularly water, can considerably influence the yield of extraction. Taking into account that naturally occurring processes take predominantly place under dynamic conditions, recent trends have been focused on the development of alternative methods aimed at mimicking environmental events more correctly than their classical batchwise static operational and sequential extraction counterparts [12].

A comprehensive survey of these developments in assessment of bioaccessible trace metal fractions in airborne particulate matter is recently reported in a review [13]. Dynamic extractions procedures provide a continuous flow of the leaching agent through the sample; thereby, soluble species are dissolved and subsequently detected offline or online providing information about the kinetics of the leaching process. The risk of sample losses as well as contamination is reduced since troublesome and time-consuming sample handling steps of batchwise procedures such as sample shaking, filtration, or centrifugation can be avoided. In literature, dynamic extraction procedures based on the concepts of stirring chambers, rotating coiled columns, packed microcartridges, and flow-through microdialysis are used mostly for the analysis of samples like fly ash, rock, soil, and sediments [12, 14]. Application of these procedures to fractionation studies (e.g., [15]) is limited because airborne particulate matter, unlike soil, sediments, and fly ash, is only available with very limited sample amounts collected on filters, typically at submilligram to milligram level [11]. To overcome problems associated with this special kind of sample, various approaches were introduced [16–18]. Instead of whole sample only aliquots of the sample in the form of either circular parts or punches of the sampling filter substrate with homogeneously distributed particulate matter were used, which enables loading of sample into the extraction unit.

Another group of methods for determination of the water-soluble fraction of metals in atmospheric aerosols can be based on the continuous sampling of atmospheric aerosols into deionized water [10, 19]. By using the aerosol growth technology, saturated water vapor condenses on the PM forming droplets, which can be collected in various impactors. The sampled particles are leached in interaction with condensed water in time, so that the soluble fraction of elements can be subsequently determined after separation of insoluble part of particles by suitable high-sensitive detection technique.

A new approach, the diffusive gradients in thin film (DGT) technique [20], has been developed to assess the bioavailability of metals in environmental systems such as sediments and soils [21]. DGT technique can measure the fluxes of labile species in water, soil, and sediment compartments in situ under undisturbed conditions and without pretreatment. In the uptake process, metals diffuse from pore water through the diffusion layer to the binding phase, typically Chelex 100 resin gel [20, 22], which is selective to transition metals. As in an organism uptake, the DGT uptake reduces metal concentration in the aqueous phase in the vicinity of the DGT probe, so that it induces resupply of metals associated with the solid phase. Thus, DGT can measure the metal fraction that is accessible from the complete metal pool in the solid phase system [21, 23].

Interpretation of DGT-induced fluxes indicates the degree of depletion of the solution metal concentration in the vicinity of the DGT probe (as determined by the thickness of the diffusive layer), the kinetics of desorption of metals, and the extent of the pool(s) of labile metal species in the particulate phase [24, 25].

The scope of this study was to investigate the kinetics of metals resupplied from an urban particulate matter into the aqueous phase by employing the DGT technique. This approach represents a useful tool in estimation of the water-soluble, mobile fraction of metals in particulate matter. Until now, to the best of our knowledge, no results have been reported concerning utilization of the DGT technique for characterization of atmospheric particulate matter in direct contact of the probe with the moisturized PM, providing information on the metal contamination and on the kinetics of metal release.

## 2. Materials and Methods

### 2.1. Particulate Matter Samples

Within a period of years 2006–2016, the total suspended particulate matter (TSPM) was sampled in annual service intervals from filters of an air-conditioning unit in the building of the Institute of Analytical Chemistry of the CAS, v.v.i., in Brno, which is situated at the heavy traffic area on the Veveri street, Brno, Czech Republic, GPS location: 49°12′27.958″N, 16°35′27.845″E. In this study, the sample collected within the period of March 2008 to February 2009 was characterized in more detail. Concentration of the PM 2.5 fraction was found [26] within the range of 20–32 *µ*g·m^−3^ for this sampling site.

### 2.2. Preparation of DGT Probes

The polyacrylamide hydrogels used in this study were prepared according to the procedure developed by Zhang and Davison [20]. Gel solution contained 15% by volume of acrylamide (Sigma-Aldrich, Germany) and 0.3% by volume of patented agarose cross-linker (DGT Research, Ltd., Lancaster, United Kingdom). *N*,*N*,*N*′,*N*′-Tetramethylethylenediamine (TEMED; Sigma-Aldrich) was used as catalyst. A freshly prepared solution of ammonium peroxydisulfate (10%, Sigma-Aldrich) was used as an initiator for polymerization. For preparation of polyacrylamide diffusive gels (APA), 10 *μ*L TEMED, and 28 *μ*L ammonium peroxydisulfate solutions were added to 4 mL gel solution. This solution was immediately cast between two glass plates separated alternatively by a plastic spacer of 0.3, 0.5, 1.0, 1.5, and 2.0 mm thickness, respectively, and allowed to set in an oven at 42–46°C for 60 min. The gel was then removed from glass plates and hydrated in ultrapure water at least for 24 h before use. Ultrapure water was exchanged three times during hydration period. Finally, the gels exhibited a stable thickness of 0.45, 0.80, 1.6, 2.35, and 3.2 mm (tolerance ± 0.01 mm), respectively, according to spacers used. Gel discs of 25 mm in diameter were cut from hydrated gel strips using a round plastic knife and then stored in 0.01 mol·L^−1^ sodium nitrate solutions at room temperature.

The resin gel consisted of 0.5 g of Chelex 100 ion-exchange resin (Na form, 100–200 wet mesh; Bio-Rad Laboratories, USA) in 2.5 mL of the gel solution. After one-day swelling of the resin in the gel solution, 3.75 *μ*L TEMED and 15 *μ*L ammonium peroxydisulfate solutions were added. Contrary to preparation of the diffusive gel, a half amount of the initiator was added to the mixture to prolong the setting time and consequently to allow settling of the resin beads on one side of the gel. Discs of resin gels were cut by the same way as diffusive gels and then stored in ultrapure water at 4°C. They reached a thickness of 0.44 ± 0.01 mm.

The DGT sampling probes were assembled by inserting the resin gel inside the piston-type DGT probe (R-SDU, DGT Research Ltd., Lancaster, UK) with resin beads oriented towards the sampler exposition window. Afterwards, the resin gel was covered with the diffusive gel and a wet protective membrane filter (0.45 *μ*m in pore size, 25 mm in diameter, and 0.13 mm in thickness (Supor®-450, Pall Corporation, Michigan, USA)), both components representing the overall diffusive layer thickness (*∆g*). Finally, the DGT probes were carefully closed. Blanks of resin gels were treated in the same way including probe preparation.

### 2.3. Deployment Experiments

The sample of PM taken from the textile filter of the air conditioner was gently stirred in order to prepare sufficient amount of homogeneous representative model sample for comparative experiments. A mixture of 20 g PM and 25 g water was equilibrated at laboratory temperature of 23°C for 24 h. The set of three DGT probes packed with polyacrylamide diffusive and Chelex 100 resin gels were gently immersed into the surface of the PM suspensions, making sure that there were no air bubbles between the PM slurry and the exposition window of the DGT probe. The probes with diffusive gel thicknesses of 1.6 mm were deployed at constant room temperature of 23°C for various time periods (short term up to 24 hours, long term up to 60 days). DGT devices were then retrieved from the PM suspension and rinsed with ultrapure water to remove all the particles adhered on the filter membrane. Analogously, the probes with diffusive gels of different thicknesses (0.45, 0.8, 1.6, 2.35, and 3.2 mm, resp.), that is, DGT probes with specific decreasing demand, were also used in a series of experiments in duration of 8 h of deployment to measure responses of PM.

### 2.4. Apparatus and Sample Analysis

In the analysis of the PM sample, pore water solution was collected from experimental suspensions by centrifugation in plastic tubes for 30 min. The supernatant was filtered through a 0.45 *µ*m membrane filter Supor-450 to remove colloidal components, then stabilized by addition of nitric acid, and stored at 4°C before analysis.

Prior ICP MS analysis, resin gels (0.24 g) and samples of PM (0.1 g) were digested in a mixture of concentrated subboil grade acids consisting of 2 mL·HNO_3_ and 0.5 mL·HCl. The samples were treated in precleaned PTFE (110 mL) reaction vessels by using focused microwave (MW) energy in one-stand closed high-pressure autoclave unit (UniClever, Plazmatronika, Wroclaw, Poland). In the first predigestion step, the sample aliquot was left to react at laboratory temperature for 5 min. Subsequently, it was heated in three steps, each in duration of 5 min, applying 70, 90, and 100 percent of microwave power (150 W) controlling working pressure within limits of 3.5/3.2, 4.0/3.5, and 4.5/4.2 MPa, respectively. After cooling down period of 10 min, the digests were diluted with ultrapure water to the final mass of 10 g. To achieve complete decomposition of PM samples inclusive silicates, 0.5 mL HF (Suprapur grade, Merck, Darmstadt, Germany) was also added to the mixture of subboil grade nitric and hydrochloric acids. Blank samples of acids (*n* = 4) and resin gels (*n* = 6) were processed analogously.

Ultrapure water, prepared by Ultra Clear system (SB Barsbüttel, Germany), was exclusively used throughout the work. The subboil grade HNO_3_ and HCl were obtained by purifying concentrated analytical reagent grade acids (Penta, Prague, Czech Republic) in the subboil quartz distillation system model MSBQ 2 (Maasen, Eningen, Germany).

Inductively coupled plasma mass spectrometry was applied as a multielement, high-sensitive technique in the determination of metals in sample solutions. An Agilent 7700 Series ICP MS with MicroMist Nebulizer was employed under operating conditions summarized in Table 1. The Octopole Reaction System (ORS) of the 7700 ICP MS was operated in helium collision mode (He mode) to exclude potential isobaric interferences according to the US EPA Method 6020A, validated by multilaboratory studies [27]. Possible spectral overlaps were checked by matrix interference and recovery tests [28]. For these tests, a series of synthetic-matrix samples and standards were prepared from the certified single-element standard stock solution Astasol® (Analytika, Prague, Czech Republic) to obtain background equivalent concentrations of elements, especially for noble metals. The analysis of real PM sample solutions revealed that the matrix elements were present in solutions below the critical interfering concentrations to cause spectral overlaps when using the He mode of the collision cell. Potential overlaps on ^103^Rh^+^ by ^87^Sr^16^O^+^, on ^105^Pd^+^ by ^89^Y^16^O^+^, and ^179^Hf^16^O^+^ on ^195^Pt^+^ were not found. When applicable, flame and electrothermal atomic absorption spectrometry was also applied to verify the results. In these comparative measurements, the bias of results was below 10% of relative standard deviation of values found by ICP MS.

The multielement calibration standards for multipoint (level) calibration were prepared by mixing of certified Astasol standards. For fast screening of the digested PM, the semiquantitative analysis mode of ICP MS was applied. In this instance, a one-point calibration was used, in which a single multielement calibration standard (1 *µ*g·L^−1^ Li, Mg, Co, Ce, Y, Tl + 10 *µ*g·L^−1^ As) was introduced before sample analysis to update the response factors.

The level of overall blanks related to analysis of PM samples and to analysis of DGT resin disks was negligible. Except platinum group metals, the blank level was 4 to 5 orders of magnitude lower than that of sample solutions, so that it could be omitted in evaluation of results. Based on 3-sigma criterion of blank fluctuation, limits of detection of selected traffic-related elements of 23 ng·L^−1^ Cd, 3 ng·L^−1^ Co, 55 ng·L^−1^ Cu, 90 ng·L^−1^ Mo, 74 ng·L^−1^ Ni, 14 ng·L^−1^ Pb, 11 ng·L^−1^ Pd, 0.14 ng·L^−1^ Pt, 0.05 ng·L^−1^ Rh, 26 ng·L^−1^ Sb, and 7 ng·L^−1^ V in aqueous media (supernatant, PM digest) and 0.13 ng Cd, 0.07 ng Co, 1.1 ng Cu, 2 ng Mo, 8 ng Ni, 0.82 ng Pb, 0.56 ng Pd, 0.002 ng Pt, 0.001 ng Rh, 14 ng Sb, and 0.22 ng V in DGT resin gel disks were achieved, respectively.

## 3. Results and Discussion

### 3.1. Characterization of the PM Sample

Particulate air pollution constitutes a complex mixture of particles, present in the atmosphere as solids or liquids that vary in mass, number, size, shape, surface area, chemical composition as well as reactivity, acidity, solubility, and origin [2].

Content of the inorganic fraction in the PM sample was assessed by charring approximately 1 g aliquots of the dry PM in ambient air atmosphere in three steps at 200°C for 1 h, 500°C for 3 h, and 900°C for 5 h. In this instance, residual ash represented 57.9 ± 0.5% (*n* = 3) of the original sample mass. The organic fraction of the PM contained probably some parts of plants, pollen species, common organic compounds as saccharides, organic acids, PAH, and high -molecular-weight alkanes [29].

Preliminary examination of the PM sample was performed by using a semiquantitative mode of ICP MS in which content of up to 80 elements could be estimated. The main components of the PM sample were as follows: Al (7.5%), Fe (4.2%), K (1.4%), Mg (0,75%), Na (0.59%), Zn (0.40%), Ti (0.25%), Ba (0.08%), and Mn (0.06%). The metallic elements Ca, Mg, Al, Si, Fe, and Mn are typical markers for soil and resuspended dust sources in the atmosphere [30]. Crustal and other elements were obviously the major contributors to composition of the PM. The results of conventional ICP MS determination of the total content of selected minor, traffic-related elements (Cd, Co, Cu, Mo, Ni, Pb, Pd, Pt, Rh, Sb, and V) are summarized in Table 2. Platinum group elements were found at similar concentration ratio as in German urban areas [3].

In natural media, metal contaminants undergo reactions with water, ligands dissolved in it, and with surface sites of the solid material. The metal partitioning is usually characterized by distribution coefficient, which is the ratio of adsorbed metal concentration (mg·kg^−1^) to the dissolved metal concentration (mg·L^−1^) at equilibrium [31]. In this work, the mobilization release of metals into water under static conditions is described by the reciprocal value, that is, by elution distribution coefficient *K*_*d*_, which reflects the metal distribution between the aqueous phase and the PM during leaching of samples with water. The *K*_*d*_ values were evaluated (Table 2) from the pore water metal concentration (mixture of 20 g PM and 25 g water in equilibrium, phase ratio *r*∼1.25) and the content of metal in the PM (solid phase). The values differ among individual traffic-related metals, decreasing exactly in the following order: Cd > Sb > Cu > Pd > Mo > Co∼Ni > Pb∼V∼Rh > Pt. These differences might reflect the origin and the speciation of individual selected metals.

### 3.2. DGT Experiments

The basic theory of DGT technique assumes that transport in both the diffusion layer of the thickness *∆g* (the thickness of polyacrylamide gel and filter membrane) and in the pore water of the slurry of PM was solely driven by molecular diffusion and that all labile metal species in the pore water had a single self-diffusion coefficient *D* that is related to free metal ions. If the binding of a metal to the resin is strong and fast in relation to the transport rate of metal species to the resin layer by diffusion, the average flux *F*, related to the unit area of the resin during the deployment, is given by the following equation [20, 22]:

(1) $$\begin{array}{c} F=\frac{M}{\left(At\right)}=\frac{Dc}{\Delta g}, \end{array}$$

where *M* is the mass of the metal trapped (metal uptake) in the resin (mol), *A* is the area of the DGT exposition window (cm^−2^) and *t* is the deployment time (s), *D* is the diffusion coefficient (cm^2^·s^−1^), *∆g* is the thickness of diffusion layer (cm), and *c* (mol·L^−1^) is the metal concentration in the pore water solution at the sampling window of the probe. This equation can be applied in evaluation and interpretation of DGT-measured data, in assessment of kinetic phenomena of metal release from the PM.

**Figure 1:**
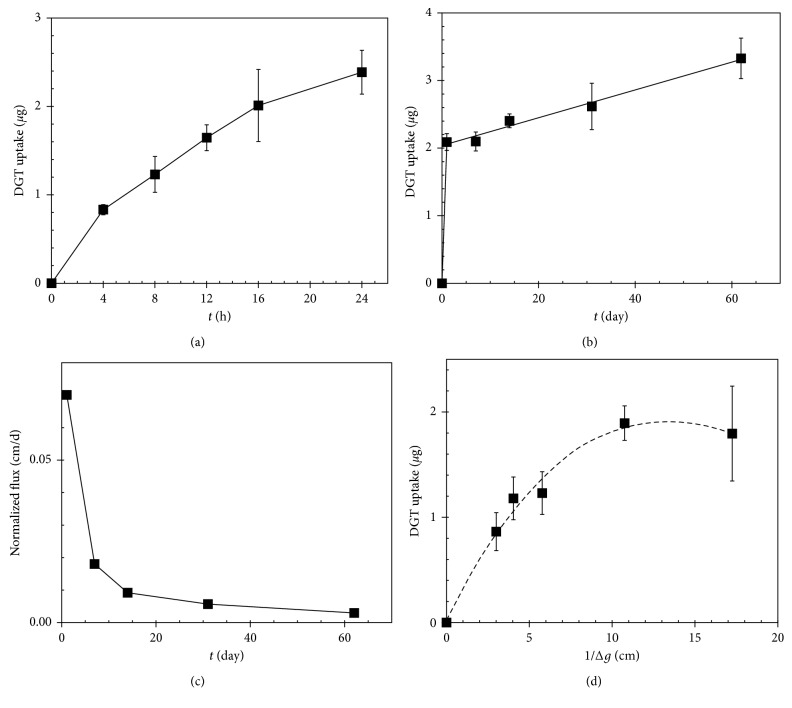
Mass of Pb found in the resin gel of the DGT probe immersed in the PM suspension (*n* = 3) for short (a) and long (b) deployment time periods (*∆*g = 1.73 mm). The metal flux into the DGT probe (c) is normalized for the metal concentration in the pore water solution. The effect of the diffusive layer thickness (8 h), representing the response of PM to the DGT probe demand, onto Pb uptake is also depicted (d).

The plots of DGT-measured uptake and flux of lead versus deployment time (Figures 1(a)–1(c)) are presented as examples of results of DGT experiments. Similar results were obtained also for other monitored metals listed in Table 2. The DGT uptake sharply decreases during a short deployment period of time. After few hours of deployment, the resupply rate from the solid phase to the solution is evidently too slow to sustain metal concentration in the aqueous phase, that is, to follow the demand of the DGT probe. Consequently, the metal flux into the DGT probe asymptotically approaches zero (Figure 1). Based on the overall concentration of a metal in the aqueous phase, uptake yields below and close to only one percent of the nominal value were recorded by the DGT probes. This observation documents that diffusive processes from the PM slurry are predominantly responsible for the resupply of metals into the solution adjacent the DGT probe [32]. This phenomenon was also reflected by the effect of the demand of the DGT probe, controlled by the diffusive gel thickness *∆g* (Figure 1), even for a very thick diffusive layer thickness of 3.33 mm. Initial DGT-measured resupply fluxes of Cd, Co, Cu, Mo, Ni, Pb, Pd, Pt, Rh, Sb, and V reached values of 3.7, 8, 160, 2.7, 27, 4, 1.1 × 10^−2^, 1 × 10^−5^, 1.1 × 10^−3^, 8.4, and 1.9 nmol·cm^−2^·day^−1^, respectively.

Generally, two metal pools associated with particles could be recognized, that is, a labile pool characterized by weak interactions (high solubility fraction) and an immobile, inert pool (insoluble fraction) incorporated strongly in the solid phase of the PM. Similar dissolution behavior was also observed for some elements in natural and anthropogenic particulate matter samples [15].

The general consensus is that ionic, water-soluble metal forms are the most bioavailable species, having the potential to access cells and organs of biota. Solubility and mobility (mobile metal fraction) of individual traffic-related metals are presented in Table 2. As documented by these data and results of DGT experiments (Figure 1), the soluble fraction of the metal pool is released within a very short time period, that is, within preparation of aqueous PM suspension and its equilibration period of one day. It is interesting to note that overall solubility of metals differs among elements of interest. It exceeded 30% of the total metal content of Cd and Sb, whereas solubility of other elements did not reach 10%. The residual fraction of the metal pool was almost insoluble. The kinetics of the subsequent release of metals was very slow even within the period of several months.

Particulate matter is a complex, heterogeneous mixture. It encompasses many different chemical components and physical characteristics. Many of them have been cited as potential contributors to toxicity [33]. Each component might have multiple sources. Therefore, identification and quantification of specific components or source-related mixtures represent one of the most challenging areas of environmental health research. The main source of PM in urban areas is the road transport [2]. Consequently, the metals of interest are divided into the source categories. It is evident from the comparison of the data in Table 2 that low content and minor soluble fraction do not represent low mobility, as documented for elements Co, Pd, and Rh. In this instance, mobility of elements can be expressed by the DGT-measured mobile fraction of the leachate, presented in Table 2, within the first stage of deployment experiments, for example, by the slope of a tangent in Figure 1. This mobile fraction reaches approximately 31, 26, and 54% of the total soluble fraction of Co, Pd, and Rh in aqueous leachate, respectively.

**Figure 2:**
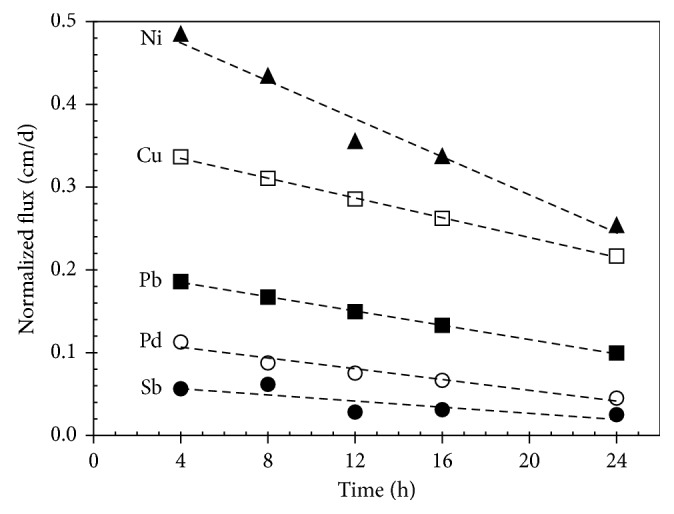
Comparison of normalized metal fluxes of Cu, Ni, Pb, Pd, and Sb into DGT probes during short-term deployment experiments (*∆g* = 1.73 mm; *n* = 3).

Resupply fluxes can be estimated from the short-term DGT experiments, from relation of calculated metal flux into the DGT probe and duration of deployment period. Calculated metal fluxes should be normalized [20] by the metal pore water concentration, as shown in Figure 2, in order to make comparison of these relations possible. Quasilinear relationship was found between the flux and the deployment time for most of the metals investigated. This decrease of the normalized flux (Table 2), expressed by the slope of the linear function, reflects depletion of a pool capacity of the mobile metal fraction with duration of deployment. The capability of resupplying metal is low when the slope is high. The slope of these lines decreased in the following order: Co, Ni, V, Rh, Cd, Cu, Mo, Pb, Pd, and Sb. In addition, the level of the normalized flux in Figure 2 represents the mobile fraction of the metal pool. In this graph, it is reflected by the virtual intercept of the line with ordinate axis. These plots can provide very interesting characteristics of PM with respect to behavior of individual elements in interaction with aqueous phase. Significant differences can be found among the elements investigated. For example, when comparing results presented in Figure 2 with the data in Table 2, they show for Sb total content of 104 *µ*g·g^−1^ Sb, relatively high solubility (32.8%), very low mobility of soluble species (1.8%), and relatively well-balanced resupply kinetics (buffering capacity) both latter assessed by DGT experiments. On the other hand, results for Pb exhibit higher total content of 411 *µ*g·g^−1^·Pb, low solubility (2.3%), higher mobility (5.7%), and slower resupply kinetics.

Similar results were also obtained in series of experiments for other PM samples taken within a period of years 2006–2016 on the same sampling site. They are not presented in this communication, because no significant trends or changes in these data have been observed.

## 4. Conclusion

The results of aqueous leaching and simultaneous DGT experiments showed that DGT technique can be successfully applied for characterization of total suspended particulate matter taken in urban area, for characterization of transition metal soluble fractions and their dissolution kinetics. DGT probes deployed in the moisturized PM disturb metal concentration close to the sampling window of the probe, so that the DGT-measured data can monitor for the response of the PM, that is, for the kinetics of metals release from the solid phase. The study of mobilization of important metals related to traffic, to various car components and fuel, showed considerable differences in metal fractionation, that is, in their content and solubility, in mobility of soluble species. DGT resupply experiments revealed that most metals in the particulate matter from Brno city were present in two fractions, in a very soluble mobile one and in an almost insoluble immobile one. This observation is in contrast to the typical behavior of soils, which exhibit more balanced resupply fluxes, that is, higher buffering metal pools for deployment time periods of several days [34, 35].

In this work, homogenized PM, representing annual accumulation on large textile fiber filters in air-conditioning system, were taken for pilot model DGT experiments on particulate slurries. Nevertheless, based on these results, the DGT technique can be successfully applied in future in a modified arrangement for measurements with routinely used filters, for example, nitrate cellulose disks of the relevant diameter, for monitoring purposes, for assessment of soluble, mobile fractions of toxic elements in aerosols of interest.

## Figures and Tables

**Table 1:** Operating conditions applied in the analysis of samples by the Agilent 7700 ICP MS instrument.

Parameter	Value
Plasma mode	Normal
RF power	1600 W
Sampling depth	7 mm
Carrier gas flow	0.6 L/min
Dilution gas flow	0.55 L/min
Spray chamber temperature	2°C
Extraction lens 1	0 V
Extraction lens 2	200 V
He cell gas flow rate	4.8 mL/min
Internal standards (online addition)	^45^Sc, ^209^Bi
Monitored isotopes	
** **In semiquantitative mode	^7^Li, ^9^Be, ^23^Na, ^24^Mg, ^27^Al,^39^K, ^45^Sc,^47^Ti, ^51^V, ^52^Cr, ^55^Mn, ^56^Fe, ^59^Co, ^60^Ni, ^63^Cu, ^66^Zn, ^75^As, ^78^Se, ^87,88^Sr, ^89^Y, ^95^Mo, ^107^Ag, ^111^Cd, ^115^In, ^118^Sn, ^121^Sb, ^137^Ba, ^140^Ce, ^179^Hf, ^205^Tl, ^206^Pb
** **In conventional analytical mode for selected traffic-related elements	^51^V, ^59^Co, ^60^Ni, ^63^Cu, ^95^Mo, ^103^Rh, ^105^Pd, ^111^Cd, ^121^Sb, ^195^Pt, ^206^Pb

**Table 2:** Characteristics of traffic-related metals in the PM listed according to source categories.

Origin/element	Total content (*µ*g·g^−1^)	Soluble fraction (%)	*K*_*d*_ (g·mL^−1^)	DGT-measured mobile metal fraction (%)	Change of the DGT-measured resupply rate (%)
	(*n* = 3)	(*n* = 5)	(*n* = 10)	(*n* = 3)	(*n* = 5)
			Mean ± SD		
*Disk brake*					
Cu	625 ± 12	9.9 ± 0.5	0.079 ± 0.004	13.4 ± 2.4	14.3 ± 0.2
Sb	105 ± 16	32.8 ± 1.2	0.263 ± 0.010	1.8 ± 0.2	4.4 ± 1.7
*Car body*					
Cd	10.1 ± 0.2	38.3 ± 2.5	0.306 ± 0.021	9.0 ± 1.8	18.7 ± 5.8
Co	30.6 ± 0.8	4.4 ± 0.2	0.035 ± 0.002	31.0 ± 3.3	56.9 ± 18.2
Ni	146 ± 4	4.3 ± 0.2	0.034 ± 0.002	22.6 ± 2,6	27.5 ± 2.8
Pb	411 ± 8	2.3 ± 0.2	0.019 ± 0.002	5.7 ± 1.2	10.3 ± 0.1
*Power fuel*					
Mo	53.9 ± 0.4	7.0 ± 0.3	0.056 ± 0.002	5.8 ± 0.5	12.1 ± 2.7
V	30.4 ± 0.7	2.3 ± 0.1	0.018 ± 0.001	17.4 ± 2.1	23.6 ± 7.2
*Catalytic converter* ^a^					
Pd	0.186 ± 0.004	8.8 ± 0.1	0.0700 ± 0.0004	26.0 ± 8.2	7.7 ± 0.9
Pt	0.016 ± 0.002	0.22 ± 0.02	0.0017 ± 0.0001	<20	n.d.^b^
Rh	0.034 ± 0.001	2.3 ± 0.1	0.0184 ± 0.0006	53.9 ± 3.8	19.2 ± 3.6

*Note*. Solubility is represented by the water-soluble fraction (*n* = 5) of the total metal content after 1-day extraction (*r* = 1.25 mL·g^−1^), *K*_*d*_ is the average (*n* = 10) distribution coefficient for aqueous extraction, the mobile metal fraction is the percentage of the *C*_DGT_-measured concentration from the overall metal concentration in the aqueous phase for the lowest demand of the probe (*∆g* = 3.33 mm, 8 h, *n* = 3) applied in the DGT experiment, and change of DGT-measured resupply rate is the percentage decrease of the metal flux per day within one-day short-term experiment (*∆g* = 1.73 mm, *n* = 5). *n*: number of replicates in evaluation of data from series of experiments; ^a^limits of detection (LOD) of 11 ng·L^−1^ Pd, 0.14 ng·L^−1^ Pt, and 0.05 ng·L^−1^ Rh for determination of these metals in aqueous media (supernatant, digest) and 0.56 ng·Pd, 0.002 ng·Pt, and 0.001 ng·Rh in DGT resin gel disks were achieved, respectively; ^b^n.d.: not determined.
